# Influences of Different Types of Interpersonal Synchronization on the Cooperative Behavior of Chinese Children

**DOI:** 10.3390/bs16050649

**Published:** 2026-04-26

**Authors:** Mingyue Liang, Jiaying Zheng, Qianqian Wang

**Affiliations:** College of Teacher Education, Ningbo University, Ningbo 315211, China; 2411030009@nbu.edu.cn (J.Z.); 2311030007@nbu.edu.cn (Q.W.)

**Keywords:** children’s cooperation, interpersonal synchrony, intentional synchrony, incidental synchrony, asynchronous behavior

## Abstract

Cooperation is an important influencing factor for individual morality and harmonious social development. Currently, most scholars select adult samples and adopt laboratory research methods. They have found that compared with asynchronous behavior, interpersonal synchronization (including both intentional and incidental synchronization) is significantly associated with higher levels of cooperative behavior. Does this conclusion apply to Chinese children? Childhood is a critical period for the development of cooperative abilities. Therefore, more effective educational approaches for fostering cooperation should be explored and adopted to promote children’s cooperative behaviors. This study randomly selected 193 students aged 8–11 (95 boys and 98 girls, M = 9.74, SD = 1.16) from 5 primary schools in a city. Based on a 2 (intentional synchronization, incidental synchronization) × 3 (measurement occasion) mixed design, a field experiment was conducted to explore the effects of different types of interpersonal synchronization on children’s cooperative behavior. However, the results showed that neither asynchronous behavior nor incidental synchronization significantly improved children’s cooperative behavior. However, the level of cooperative behavior under intentional synchronization conditions was significantly higher than that under incidental synchronization conditions. This characteristic may be related to China’s long-standing collectivistic education, which can help educators reflect on and optimize their cooperation education practices. This finding deserves attention from cooperation researchers.

## 1. Introduction

Cooperation refers to an agreement among two or more individuals to cooperate and support each other, aiming to achieve the maximum common benefit with minimal effort (Chen & Pang, 2001). Cooperation is an important dimension of children’s social development (Wang, 1996). There is abundant research on intervention strategies for children’s cooperative behavior, such as introducing a reputation mechanism (Kang et al., 2024); setting reasonable reward and punishment methods (Zhu et al., 2020); and selecting cooperative games instead of competitive ones (Bay-Hinitz et al., 1994), among others.

Among numerous intervention measures, interpersonal synchronization behavior is one of the research focuses. Interpersonal synchronization behavior refers to the temporal overlap of actions among two or more people (Cirelli, 2018), emphasizing the similarity of behaviors among multiple people at the same time. Studies by many scholars have shown that interpersonal synchronization behavior plays an important role in enhancing connections between people and promoting cooperation. For example, collective rituals involving similar actions can make the general public tend to share common beliefs and values ideologically, thereby enhancing the degree of unity and cooperation to achieve the common goals of the social group (Zhang, 2014). Synchronization behavior can effectively enhance the sense of connection and closeness among group members (Tarr et al., 2018), interpersonal cooperation (Wiltermuth & Heath, 2009), and the generation of altruistic emotions (Reddish et al., 2013). At the same time, the role of such synchronization behavior in enhancing members’ unity and cooperation can cross group boundaries; participants in synchronization behavior can also show greater prosociality (Reddish et al., 2016) and cooperative tendencies (Good et al., 2017) towards out-group members.

However, some studies have reported opposite results, i.e., the promoting effect of interpersonal synchronization behavior on prosociality (e.g., cooperation) is not significant. Here, prosociality refers to the psychological traits and behavioral tendencies of individuals to voluntarily benefit others, such as cooperative behavior. A study by Wallot et al. (2016) found that interpersonal synchronization did not affect heart rate measurements when assembling car models, and synchronization was negatively correlated with product quality. Another study found that participants in the dance synchronization group (involving synchronization with others and music) did not show a stronger willingness to cooperate in economic games (Tarr et al., 2016).

The above studies are from different countries and regions, and the reported effects of interpersonal synchronization on cooperative behavior also vary. It can be seen that whether interpersonal synchronization has an effect on cooperative behavior remains to be further studied in different cultural environments.

### 1.1. Types of Interpersonal Synchrony

Regarding the promoting mechanism of interpersonal synchronization on cooperative behavior, there are mainly the following two explanations. The first type of research holds that the perception of similarity in behaviors promotes people’s cooperative behavior. This is a “self-other representation overlap” model, which promotes cooperation by generating a sense of connection through the perception of similarity with others’ representations and behaviors (Ma & Cui, 2022). Self-other overlap refers to the phenomenon in which individuals in interpersonal relationships accept and understand others’ ideas and resources to varying degrees, resulting in the overlap of self and others’ information representations. The higher the degree of self-other overlap, the more obvious the helping behavior (Zhong et al., 2015). Durkheim speculated that behavioral consistency can be transformed into ideological consistency. Everyone’s thoughts are drawn into the same vortex, and individual types are almost confused with the collective consciousness (Durkheim, 2012). This blurring of the boundary between self and others may be the reason for enhancing people’s willingness to cooperate.

The second type of research holds that the similarity of behaviors is not sufficient to promote people to become more cooperative; the reason why people become more cooperative is the addition of shared intention during the synchronization process. Tomasello and Carpenter (2007) emphasized that the process of shared intentionality is very important in the early cognitive development of children. Shared intentionality enables people to trust each other and coordinate cooperation towards a common goal or belief within a common plan or context. Individuals can develop a sense of reliance on others (trust) when they are able to understand and share others’ mental states (shared intentionality). Trust represents the manifestation of shared intentionality in risky situations: when cooperation is accompanied by uncertainty (e.g., the risk of defection), shared intentionality translates into trust in peers, with the belief that others will uphold shared commitments (Schilbach et al., 2008). In turn, heightened cooperative expectations (i.e., trust) lead individuals to increase their cooperative behavior in social dilemmas (Reddish et al., 2013). Some studies have shown that synchronous rituals that strengthen the moral community and are regarded as having transcendent values will amplify prosocial judgments and behaviors (Fischer et al., 2013). Overall, existing studies speculate that synchronous behavior with shared intention can effectively promote people’s cooperative behavior.

These two types of explanations correspond to two distinct forms of interpersonal synchrony: incidental synchrony and intentional synchrony. The first type is incidental synchrony, which refers to behavioral alignment achieved through situational cues or low-level perceptual motor coupling. During this form of synchrony, although individuals’ actions are coordinated, achieving synchrony is not their explicit goal (Dong et al., 2015). Their behavioral alignment emerges unintentionally due to situational or stimulus factors—examples include coordinating movements to a metronome, naturally matching stride lengths while walking side by side, or audiences converging in their clapping rhythm. The second type is intentional synchrony, in which individuals deliberately adjust their actions to achieve alignment, driven by shared intentionality. In this form of synchrony, maintaining behavioral coordination serves as a group goal; accordingly, individuals intentionally modify their movements to align with others, in the awareness that others share the same objective (Reddish et al., 2013). Within this context, shared intentionality refers to the group goal of sustaining behavioral synchrony—that is, the instruction given to the group by the experimenter to align one’s movements with those of others around them.

### 1.2. The Impact of Interpersonal Synchrony on Cooperative Behavior

Researchers have examined the effects of these two distinct forms of synchrony on human cooperative behavior, with two main findings:

First, compared with asynchronous behavior, incidental synchrony has been shown to be associated with increased cooperative behavior. For example, Hove and Risen (2009) found that experiencing incidental synchrony enhanced participants’ sense of belonging, and those in the synchronous condition reported greater liking for the experimenter than those in the asynchronous condition. Individuals in the asynchronous state may experience a weaker sense of belonging, resulting in lower ratings of liking toward the experimenter compared with participants in the incidental synchrony condition.

Second, relative to incidental synchrony, intentional synchrony is more strongly associated with individual cooperative behavior. In an experiment with 123 volunteers aged 17–42, Reddish et al. (2013) found that intentional synchrony promoted greater cooperation among group members compared with asynchronous conditions, incidental synchrony, and passive conditions. Within group synchrony, the combination of low-level motor and perceptual systems with higher-level intentional systems elicits particularly strong cooperative responses. Dong et al. (2015) found that intentional synchrony significantly increased individuals’ conformity to others’ opinions compared with incidental synchrony and asynchronous behavior. Most of these studies employed social dilemma paradigms to observe and measure individual cooperative behavior, such as the Prisoner’s Dilemma game and the Public Goods Game. The present study also used the Public Goods Game as the measure of cooperative behavior. Operationally, cooperative behavior was defined as the extent to which an individual, after receiving a fixed number of tokens, chose to contribute a portion of them to a public account. The more tokens an individual contributed to the public account, the higher the level of cooperative behavior.

In summary, existing research suggests that incidental synchrony is associated with higher levels of cooperative behavior than asynchronous behavior and that intentional synchrony is associated with higher levels of cooperative behavior than incidental synchrony. However, most of these studies are grounded in Western individualistic cultural contexts and use adult samples, with relatively little attention paid to whether these findings generalize to Chinese children. Whether the above conclusions hold among Chinese children therefore warrants further investigation. On the one hand, for children, integrating into social groups is not only a key developmental task but also a core indicator of successful socialization. Ages 6 to 12 represent a critical period for the consolidation and expansion of children’s peer relationships (Petersen & Crockett, 1987). Psychologist Robert L. Selman emphasized that children aged 8 to 9 years view friends as collaborators and believe that friendships require cooperation, shared goals, and consistent routines. This stage is thus a critical window for fostering children’s cooperative abilities (Sun, 2017); On the other hand, it is valuable to study the role of interpersonal synchrony in promoting cooperative behavior in children. Children show friendly relationships with others or express their group identity with others through social imitation and strong consistency (Tomasello, 2017).

Exploring the promotional effects of different types of interpersonal synchronization on children’s cooperative behavior enables educators to adopt more effective educational strategies, so as to facilitate the development of children’s cooperation during this critical developmental period. Accordingly, this study adopted a field experimental design to investigate the effects of different types of synchrony on cooperative behavior among primary school children. Drawing on previous research, the following three hypotheses were proposed:

**Hypothesis** **1:** *Asynchronous behavior is not associated with significantly higher levels of cooperative behavior in children.*

**Hypothesis** **2:** *Incidental synchrony is associated with significantly higher levels of cooperative behavior in children.*

**Hypothesis** **3:** *Intentional synchrony is associated with higher levels of cooperative behavior in children compared with incidental synchrony.*

## 2. Methods

### 2.1. Participants

The study employed a 2 × 3 mixed experimental design. The between-subjects independent variable was group type (intentional synchrony group, incidental synchrony group), and the within-subjects variable was measurement occasion (pre-activity, post-activity). With an effect size (f) set to 0.25, a desired alpha level of 0.05, and statistical power set to 0.8, the minimum required sample size calculated using G*Power 3.1 was 128.

**Figure 1:**
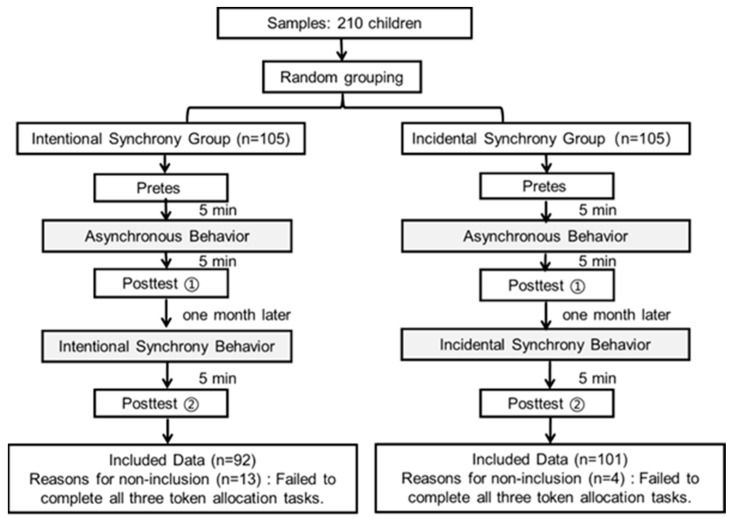
Flowchart of the Experimental Procedure. Note: n = number of participants; n = 105 indicates the initial sample size in each group; n = 92 and n = 101 represent the final included sample sizes after excluding participants who failed to complete all three token allocation tasks (n = 13 and n = 4, respectively).

Five primary schools were randomly selected in N City. From each school, one class was randomly chosen, resulting in a total of 210 children aged 8 to 11 years. The class rosters of the five classes were randomized, and children were then assigned to either the intentional synchrony group or the incidental synchrony group based on the parity of their student ID numbers, with 105 children in each group. The two groups were treated as separate new collectives and participated in the experiment independently. Some children were not fully engaged in the experiment and failed to complete all token allocations, leading to missing data in their distribution records. These cases were excluded during data entry, excluding these data had no significant impact on the final analytical results. The final valid sample consisted of 193 children (see Figure 1): 92 in the intentional synchrony group and 101 in the incidental synchrony group, including 95 boys and 98 girls (M = 9.74, SD = 1.16). All children received appropriate gifts and a participant fee after the experiment based on their performance. Upon completion of the experiment, the purpose of the study was explained to the participants, and informed consent was obtained from both the children and their guardians. This study was reviewed and approved by the Academic Committee of the College of Teacher Education, Ningbo University.

### 2.2. Experimental Materials

Previous studies have widely used the Public Goods Game as the primary measure of cooperative behavior. This paradigm involves trade-offs between self-interest and ethical considerations under uncertainty and is commonly employed in experiments on cooperation. The Public Goods Game is one of the most extensively studied social dilemmas in experimental economics (Li & Noussair, 2024) and has been frequently applied in developmental research to examine cooperation and prosociality in children (Majolo & Maréchal, 2017; Vogelsang et al., 2014). The present study adopted the Public Goods Game to measure children’s cooperative behavior, in order to examine the association between interpersonal synchrony and children’s cooperation. Following the procedure used by (Gintis, 2009), the Public Goods Game was administered as follows: each participant allocated a portion of their tokens to a public account, keeping the remainder in their private account. Participants were informed of the total number of tokens contributed to the public account, and additional tokens were added to each participant’s private account as a proportion of the total contribution to the public account.

### 2.3. Experimental Procedure

#### 2.3.1. Pre-Test

**Figure 2:**
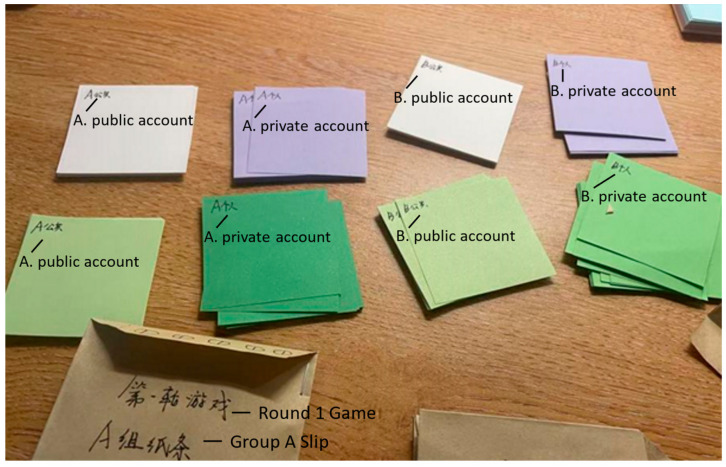
Notes Ensuring Anonymity.

A public goods game was used to measure participants’ cooperative behavior before the experiment: each participant was given 10 tokens and instructed to allocate them between their private account and a collective public account at their own discretion, where tokens kept in the private account were retained in full by the participant, and the total number of tokens allocated to the public account was averaged and distributed equally to each group member’s private account. For example, in a group of two members, if Participant A kept 6 tokens and allocated 4 to the public account while Participant B kept 5 tokens and allocated 5 to the public account, Participant A would receive 6 + (4 + 5) ÷ 2 = 10.5 tokens, and Participant B would receive 5 + (4 + 5) ÷ 2 = 9.5 tokens. To ensure anonymity during allocation, each participant received two small slips of paper: they wrote the number of tokens allocated to the public account on one slip and the number kept for themselves on the other (see Figure 2). This method also prevented participants from forgetting how many tokens they had kept for themselves. Participants folded each slip separately, and after each round, participants submitted the slip for the public account to the experimenter while keeping the private account slip for their own reference.

After the game, participants exchanged the tokens in their personal accounts for corresponding gifts with the experimenter. The exchange rules were as follows: the experimenter ranked the token amounts of all 210 participants from highest to lowest and divided them into three levels, with 70 exchange quotas for each level. If a participant’s token amount in their personal account was greater than or equal to that of the 70th participant, he or she would receive the largest gift; if a participant’s token amount was greater than or equal to that of the 140th participant but less than that of the 70th participant, he or she would receive the medium gift; if a participant’s token amount was less than that of the 140th participant, he or she would receive the smallest gift. Participants exchanged their gifts at the end of the experiment; before that, they only knew the size of the gifts but not their specific contents.

It should be noted that before the start of the game, the experimenter explained the game rules and exchange rules, wrote them on the blackboard, and then asked all participants if they understood the rules. To ensure that the children truly grasped the game rules, the experimenter presented a situational math problem, which stated: “If there are 20 students in the class, and Xiaoming puts 4 tokens into the public account and keeps 6 tokens for himself, how many tokens will Xiaoming finally get? How many tokens will each of the other 19 students in the class get?” All participants were required to answer this question independently, and a correct answer was considered as an understanding of the reward rules. After the experimenter collected the answers, they re-explained the game rules to the participants who did not understand them until all participants fully comprehended the reward rules before starting the game.

The experiment was conducted in a real classroom environment of a school, and the teachers of these participants were not present throughout the process. Before the experiment started, the experimenter told the participants: “Our main activities today are to practice a relaxation exercise and play some small games to relax our minds and bodies.” Therefore, the participants were unaware of the true purpose of the experiment. Most of them appeared relaxed and happy during the experiment, showing no obvious signs of stress. To ensure procedural equivalence between the incidental synchronization and intentional synchronization conditions, the two tasks were matched in terms of difficulty, duration, and attentional demands. Both conditions employed identical movement patterns, tempo, and total trial length. Participants in both groups were instructed to focus on the task and follow the cues, so as to minimize potential differences in motivation or attentional allocation. They were required to keep quiet during the game and not to communicate with each other about their token allocation ratios. After the game, the experimenter counted the number of tokens in each participant’s personal account and exchanged them for corresponding gifts.

To ensure that participants could focus on completing the experiment, the experimenter prepared a 5-yuan reward as compensation for each participant. This 5-yuan reward was different from the gifts exchanged with tokens; all participants who participated in and completed the experiment received this reward, and the amount was 5 yuan for everyone. The experiment proceeded smoothly, and all participants complied with the relevant rules throughout the procedure.

#### 2.3.2. Manipulation of Asynchronous Behavior

Following the experimental design of synchrony studies by Reddish et al. (2013), the experimenter instructed all participants to complete a set of actions consisting of three different simple movements, each lasting two minutes.

(1) Raise and lower their left arm up and down, followed by raising and lowering their right arm up and down (arm swinging).

(2) Swing their upper body to the left and upward, then to the right and upward from an upright position (twisting).

(3) Lift their left leg up and down, followed by lifting their right leg up and down while standing (stepping in place).

Participants were given 10 s to practice these three movements to ensure they could perform them on time. Upon the experimenter’s command of “Start,” participants executed the three actions sequentially (arm swinging, twisting, stepping), each for two minutes. The experimenter then issued a “Stop” command and instructed the participants to begin the next movement.

#### 2.3.3. Post-Manipulation Assessment (Post-Test ①)

Five minutes after completing the asynchronous manipulation, the experimenters again measured participants’ cooperative behavior using the Public Goods Game. To effectively control for carryover effects from the initial manipulation and minimize potential fatigue and practice effects, all participants did not participate in the subsequent experimental procedures until one month after completing the post-test for the asynchronous condition (see Figure 1).

#### 2.3.4. Manipulation of Interpersonal Synchrony

The movements performed in the interpersonal synchrony condition were the same as those in the asynchronous condition. Participants in the intentional synchrony group and the incidental synchrony group performed the same three movements as in the asynchronous condition, but in accordance with different requirements. The specific movement requirements were as follows:

Incidental synchrony condition: The experimenter played a beat beside the participants (with the beats per minute (BPM) set to 65) and instructed the students to complete the above movements in time with the beat.

Intentional synchrony condition: The experimenter instructed the participants to perform the three movements and make every effort to keep their movements synchronized with those of the other students around them.

During the practice of interpersonal synchrony, the experimenter was required to judge whether the participants achieved synchronization. The experiment was videotaped, and researchers from other related fields watched the recordings and judged whether participants achieved synchronization based on established criteria for synchronous behavior. The criterion for synchronization was that all participants in the group had exact rhythmic matching of actions with each other in time and phase (Mogan et al., 2017), and the synchronization of each movement had to be maintained for 2 min (Reddish et al., 2013). According to this assessment all participants met the standard and proceeded to the second post-test.

#### 2.3.5. Post-Test of Interpersonal Synchrony (Post-Test ②)

The second post-test still adopted the public goods game. After the game, all participants filled out a Cooperative Perception Scale. This scale was adapted from the study by Wiltermuth and Heath (2009), aiming to measure the psychological changes in participants after the synchrony behavior, and it is widely used in studies related to synchrony behavior (Reddish et al., 2013; Gvirts et al., 2023). The scale has also been adapted and used in other studies involving children with a mean age of 8.77 years to measure their willingness to approach each other (Tunçgenç & Cohen, 2016). The Cronbach’s α reliability coefficient of the scale was 0.786, indicating good reliability. The scale used a 7-point Likert scale to measure entitativity, perceived similarity, trust, and emotion respectively. It specifically included four questions: (1) I feel that I am a member of the same team as the other students participating in the activity. (2) I trust the other students participating in the activity. (3) I am similar to the other students participating in the activity. (4) I am happy now. (1 = “Not at all”, 7 = “Very much”).

## 3. Experimental Results

**Figure 3:**
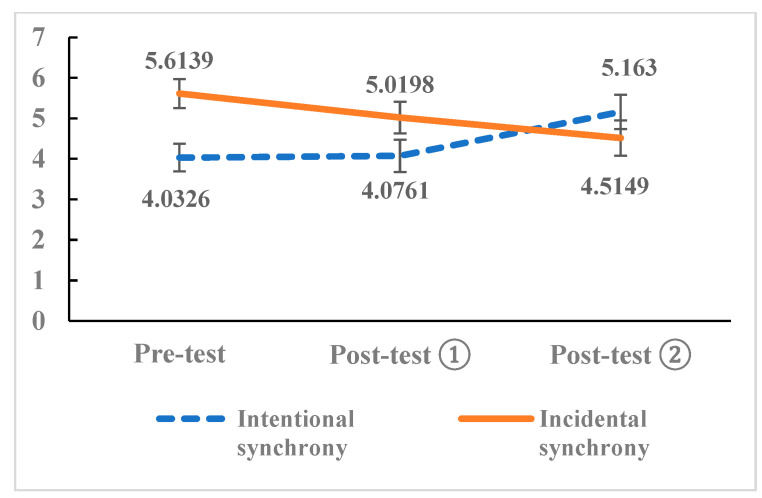
Changes in Participants’ Token Allocation Amounts. Data are presented as means ± standard error of the mean (SEM). The intentional synchrony group had n = 92, and the incidental synchrony group had n = 101. The horizontal axis represents the token allocation trails, and the vertical axis represents the number of tokens allocated. The values on the lines indicate the mean token allocation amounts for each group at the corresponding allocation trial.

The means of the three token allocation amounts for participants in both groups are presented in Figure 3. The solid line represents the mean token allocation amounts of the incidental synchrony group across the three trials, while the dashed line represents the mean token allocation amounts of the intentional synchrony group across the three trials.

The study used SPSS Statistics 23 to analyze all data. To test the interaction effects of the experiment, a mixed-design analysis of variance (ANOVA) was used to analyze the three sets of token allocation data from the experiment. Mauchly’s test of sphericity was used to test the sphericity assumption for the within-subjects factor. The results showed that Mauchly’s W = 0.886, *p* < 0.001, indicating that the sphericity assumption was not met. Therefore, the Greenhouse-Geisser correction was applied to both the within-subjects effects and interaction effects, and the corrected statistical results are reported below.

The results showed that the main effect of group was not significant, indicating that there was no significant difference in the overall number of tokens allocated between the intentional synchrony group and the incidental synchrony group. The main effect of measurement time was not significant, indicating that there was no significant difference in the overall level of token allocation across the three measurement time points. The interaction effect between group and measurement time was significant, indicating that there was a significant difference in the changing trend of token allocation amount between different groups with the increase in measurement times (see Table 1).

Since the interaction effect was significant, further tests were conducted on the simple main effect of group and measurement time. All simple effect tests adopted the Bonferroni correction method to control the Type I error of multiple comparisons.

The results of the simple main effect test of group showed that: in the pre-test, the simple main effect of group was significant, F = 10.242, *p* = 0.002, partial η^2^ = 0.051, indicating that in the pre-test, the number of tokens allocated in the incidental synchrony group was significantly higher than that in the intentional synchrony group. In the first post-test (Post-test ①), the simple main effect of group was not significant, F = 2.831, *p* = 0.094, partial η^2^ = 0.015, indicating that there was no significant difference in the number of tokens allocated between the two groups in Post-test ①. In the second post-test (Post-test ②), the simple main effect of group was not significant, F = 1.125, *p* = 0.290, partial η^2^ = 0.229, indicating that in Post-test ②, the number of tokens allocated in the intentional synchrony group was not significantly higher than that in the incidental synchrony group.

The results of the simple main effect test of measurement time showed that: in the intentional synchrony group, the simple main effect of measurement time was significant, F = 3.916, *p* < 0.05, partial η^2^ = 0.04; in the incidental synchrony group, the simple main effect of measurement time was also significant, F = 3.501, *p* < 0.05, partial η^2^ = 0.036. This indicated that regardless of the intervention of intentional synchrony or incidental synchrony, the number of tokens allocated by the participants was not stable at different measurement time points, but showed a systematic changing trend.

To clarify the pairwise differences in the number of tokens allocated within each group at different measurement time points, multiple comparisons with Bonferroni correction were conducted. The results showed that in the intentional synchrony group, there was no significant difference between Post-test ① and the pre-test (*p* = 1), Post-test ② was significantly higher than Post-test ① (*p* = 0.025), and Post-test ② was significantly higher than the pre-test (*p* = 0.039). In the incidental synchrony group, there was no significant difference between Post-test ① and the pre-test (*p* = 0.187), no significant difference between Post-test ② and Post-test ① (*p* = 0.591), and Post-test ② was significantly lower than the pre-test (*p* = 0.034). This indicated that the number of tokens allocated in the intentional synchrony group showed a significant upward trend, with no significant change from the beginning to the middle, and a significant increase in the later period, while the number of tokens allocated in the incidental synchrony group showed a significant downward trend, with slight fluctuations in the early stage but no statistical difference, and finally significantly lower than the initial level.

The results of the mixed-design ANOVA revealed that the token allocation amount in the intentional synchrony group exhibited a significant upward trend, remaining unchanged from the pre-test to Post-test ①, and showing a significant increase in Post-test ② compared to both the pre-test and Post-test ①, demonstrating a positive intervention effect. In contrast, the token allocation amount in the incidental synchrony group showed a significant downward trend, with slight fluctuations but no statistically significant difference from the pre-test to Post-test ①, and was significantly lower than the baseline (pre-test) level in Post-test ②. At baseline (pre-test), the token allocation amount in the incidental synchrony group was significantly higher than that in the intentional synchrony group. However, after the respective interventions, the between-group differences in token allocation amounts between the two groups in Post-test ① and Post-test ② disappeared, with no statistically significant differences.

Notably, the incidental synchrony group had a significantly higher baseline score than the intentional synchrony group. The absence of a difference in final scores does not equate to an absence of difference in the magnitude of change. In fact, the intentional synchrony group showed an upward trend, while the incidental synchrony group showed a downward trend; their “convergence” resulted in similar final scores. Therefore, to directly compare the magnitude of the intervention effects between the two groups, an independent-samples *t*-test was conducted on the change scores D (D = Post-test ②—Post-test ①) for both groups. Analyzing the change scores allows for a direct comparison of the intervention effectiveness across groups, which is more aligned with the core research objective (whether the intervention is effective). The results of the Levene’s test for equality of variances showed F (1, 191) = 0.268, *p* = 0.605 > 0.05, satisfying the assumption of homogeneity of variance. Thus, the *t*-test results assuming equal variances are reported (see Table 2). The independent-samples *t*-test indicated that the pre-post change score D (M = 1.09, SD = 3.68) in the intentional synchrony group was significantly higher than that in the incidental synchrony group (M = −0.51, SD = 4.12). There was a statistically significant difference in the pre-post change scores between the intentional and incidental synchrony groups, t (191) = 2.819, *p* = 0.005, 95% confidence interval [0.478, 2.706]. The effect size was calculated using Cohen’s d, which was 0.41, representing a small to medium effect (Cohen, 2013). This indicates that the improvement in token allocation amount in the intentional synchrony group was significantly superior to that in the incidental synchrony group.

By comparing the scores of the two groups on the scale, it was found that students in the intentional synchrony group scored higher on the four items of entitativity, perceived similarity, trust, and emotion (see Table 3). Compared with the incidental synchrony group, students in the intentional synchrony group showed higher levels of team belongingness, perceived similarity with peers, and trust in classmates.

Specifically, for the score on the trust item, the results of the independent-samples *t*-test showed that there was a statistically significant difference in trust levels between the intentional synchrony group and the incidental synchrony group, t (146.25) = 2.143, *p* = 0.034, 95% confidence interval [0.044, 1.094]. The effect size was calculated using Cohen’s d, d = 0.34, indicating a small effect. For the scores on the other items in the scale, although the intentional synchrony group scored higher than the incidental synchrony group, the difference did not reach a significant level. This indicates that compared with other factors such as entitativity, perceived similarity, and emotion, intentional synchrony intervention has a significantly higher correlation with trust levels, verifying the positive effect of the intervention. The trust score of the intentional synchrony group was significantly higher than that of the incidental synchrony group, which is consistent with the previous research speculation that the combination of shared intention and synchronous behavior is positively associated with higher levels of cooperative expectations and cooperative behavior (Reddish et al., 2013).

## 4. Discussion

### 4.1. Research Findings

This study aimed to investigate the impact of interpersonal synchrony on the cooperative behavior of Chinese children. Conducted in real school settings using educational experimental methods, the study found the following results:

(1) Asynchronous behavior did not produce a statistically significant difference in improving the cooperative behavior of children aged 8–11. This result is consistent with previous relevant studies, supporting Hypothesis 1. Asynchronous behavior is unlikely to exert a significant effect on improving children’s cooperative behavior.

(2) Children aged 8–11 in the incidental synchrony condition did not show significant differences in cooperative behavior. This result differs greatly from previous studies, leading to the rejection of Hypothesis 2. This finding implies that incidental synchronization may hardly significantly enhance cooperative behaviors among Chinese children.

(3) The cooperative behavior of children aged 8–11 in the intentional synchrony condition was significantly higher than that in the incidental synchrony condition, supporting Hypothesis 3. Given that the trust scores of the intentional synchronization group were significantly higher than those of the incidental synchronization group, we infer that shared intentionality combined with behavioral synchronization contributes to enhancing children’s cooperation expectations and thereby their cooperative behaviors. This inference is consistent with previous research, which suggests that collectivistic cultural values may constitute an indispensable component of the mechanism through which synchronization elicits cooperation (Reddish et al., 2013).

In summary, the results indicate that synchronous behavior accompanied by shared intention is more likely to enhance children’s cooperative behavior. However, mere synchronous physical movements may have a weak promoting effect on children’s cooperative behavior, making it difficult to induce significant changes in their cooperative behavior. This finding differs from many previous results obtained in Western countries.

Why might incidental synchronous behavior be difficult to enhance the cooperative behavior of Chinese children? This may be related to cultural backgrounds between China and Western countries. Relevant studies have also speculated on cultural background as an influencing factor, finding that synchronous movement can promote cooperation more effectively among participants from different sociocultural backgrounds, but this phenomenon is not observed among participants with the same sociocultural background (Cross et al., 2019). This may suggest that the promoting effect of interpersonal synchronization on cooperative behavior may be masked in some cases; when there is an obvious and strong social connection between synchronized individuals, the prosocial effect of synchronization may be less significant (Ma & Cui, 2022). However, such speculation lacks direct observational evidence in the present study and only provides a plausible interpretation for the current findings. Consistent with previous research, the present study found that children’s cooperative behavior was significantly higher in the intentional synchronization condition than in both the asynchronous and incidental synchronization conditions.

### 4.2. Research Significance

Based on the above, the present study explores the intrinsic association between children’s interpersonal synchronization and cooperative behavior from a theoretical perspective, while also providing novel practical insights for cooperation education in children. The findings help educators in Chinese schools reflect on and optimize their conventional approaches to cooperation education, thereby promoting the development of cooperative behaviors among Chinese children. The experimental results of this study offer a new perspective for children’s cooperative education. In the context of Chinese culture, if educators aim to promote children to exhibit more cooperative behavior, establishing a shared intention for them within the collective may be a more effective approach. This can enhance their trust in group members, increase cooperative expectations, and thereby promote cooperation. However, without the inclusion of a shared intention, simply engaging children in more collective activities and maintaining behavioral synchrony with their peers may hardly lead to a significant improvement in their cooperative behavior. However, given the methodological limitations of this study, the above inferences need to be further examined and refined in future empirical research.

However, the study still has several limitations. For instance, the discussion regarding cultural background is only a conjecture based on previous relevant studies, and the research did not directly measure or examine the children’s cultural tendencies. Although the study established a shared intention for the intentional synchrony group, it did not directly measure students’ perception of and attention to the shared intention using a scale. Therefore, we cannot fully confirm that the established shared intention effectively cultivated children’s focus on the collective’s common goals. Additionally, we cannot definitively conclude that the shared intention we established caused children’s cooperative behavior; we can only speculate, based on the results of this study, that the establishment of a shared intention may prompt children to exhibit more cooperative behavior.

Furthermore, our experimental design was indeed subject to order effects, fatigue, and practice effects. Even with a one-month interval between experimental sessions, these effects still persisted and were difficult to resolve through data analysis. The sample selection exhibited a classroom clustering effect, as students were clustered within classes, which may have influenced statistical estimates. The absence of a genuine control condition meant that the operationalization of incidental and intentional synchronization might still have been confounded by differences in attention, motivation, and task demands. The generalizability of the findings was limited due to the sample’s geographic and age restrictions. Baseline differences between groups also remained a concern, and using deviation scores only partially addressed this issue. The measurement tools were similarly limited, as no explicit instrument was used to assess participants’ synchronization behavior. Future research will further elaborate and refine this line of inquiry to obtain clearer, more robust, and systematic results.

Future research can conduct more studies on the internal mechanisms by which intentional synchrony and incidental synchrony promote cooperation, and carry out experiments across different cultural backgrounds. This may lead to a deeper understanding of the different roles of incidental synchrony in social connections at different levels.

## Figures and Tables

**Table 1:** Results of Main Effects and Interaction Effects in Mixed-Design Analysis of Variance.

Source of Variance	*F*(df_1_, df_2_)	*p*	Partial η^2^	Effect Size Grade
Group (Between-Subjects Main Effect)	*F*(1, 191) = 1.875	0.173	0.010	Small Effect
Measurement Time (Within-Subjects Main Effect, GG Corrected)	*F*(1.77, 338.67) = 0.700	0.482	0.004	Small Effect
Group × Measurement Time (Interaction Effect, GG Corrected)	*F*(1.77, 338.67) = 8.619	<0.001	0.043	Small Effect

**Table 2:** Results of Independent-Samples *t*-test for Pre-Post Synchrony Differences Between the Two Groups.

Group	n	M	SD	t(191)	*p*	95% CI	Cohen’s d	Effect Size
Intentional Synchrony	92	1.0870	3.6784	2.819	0.005	[0.478, 2.706]	0.41	Small to Medium Effect
Incidental Synchrony	101	−0.5050	4.1246					

**Table 3:** Scale Scores of the Two Groups.

	Group	M	SD
Trust	Intentional Synchrony	6.0137	1.38938
	Incidental Synchrony	5.4444	1.89077
Entitativity	Intentional Synchrony	6.3288	1.30229
	Incidental Synchrony	5.9012	1.59378
Perceived Similarity	Intentional Synchrony	5.2466	1.76211
	Incidental Synchrony	4.9012	1.89476
Emotion	Intentional Synchrony	5.9726	1.68302
	Incidental Synchrony	5.8025	1.77778
Total Scale Score	Intentional Synchrony	23.5616	4.51229
	Incidental Synchrony	22.0494	5.80496

## Data Availability

The data presented in this study are available on request from the corresponding author.
